# Characterisation and tanning effects of purified chestnut and sulfited quebracho extracts

**DOI:** 10.1186/s42825-024-00171-9

**Published:** 2024-09-02

**Authors:** Silvia Conca, Vanessa Gatto, Riccardo Samiolo, Samuele Giovando, Andrea Cassani, Elisa Tarabra, Valentina Beghetto

**Affiliations:** 1 Crossing S.R.L., Viale Della Repubblica 193/B, Treviso, 31100 Italy; 2 CRCF Srl for Silvateam Spa, Via Torre 7, San Michele Mondovì, 12080 Italy; 3 Department of Molecular Sciences and Nanosystems, University Ca’ Foscari of Venice, Via Torino 155, Mestre, 30172 Italy; 4 Consorzio Interuniversitario Per Le Reattività Chimiche E La Catalisi (CIRCC), Via C. Ulpiani 27, Bari, 70126 Italy

**Keywords:** Purification, Chestnut, Quebracho, Vegetable tanning, Biomass, Leather

## Abstract

Vegetable tannins are environmentally friendly tanning agents. However, they generally impart a dark colour to the tanned leather and highly contribute to the organic load in wastewaters. In this study, we employed a purification protocol separately on chestnut tannin (CT) and sulfited quebracho tannin (QT) to obtain the purified fractions (PCT and PQT). These samples were characterised by GPC, ^1^H NMR, ^13^C NMR, FT-IR, and HPLC–DAD techniques and applied for tanning tests. Through the purification process, non-tannin components and smaller molecules such as gallic acid, glucopyranose, and catechin were effectively removed from CT and QT, which consequently led to the reduced moisture content, pH value, and lighter colour of purified fractions. The crust leathers processed with PCT and PQT showed desirable light shades. Moreover, the organic loads in PCT and PQT tanning wastewater were reduced by 13.5% and 19.1%, respectively, when compared to those in traditional CT and QT tanning wastewater. Additionally, the physical and mechanical characteristics of crust leathers processed with PCT and PQT were comparable to those processed with CT and QT. Thus, purification of vegetable tannins may serve as a feasible strategy for producing light-colored vegetable-tanned leather while minimizing organic pollutant discharge during the vegetable tanning process.

**Graphical Abstract:**

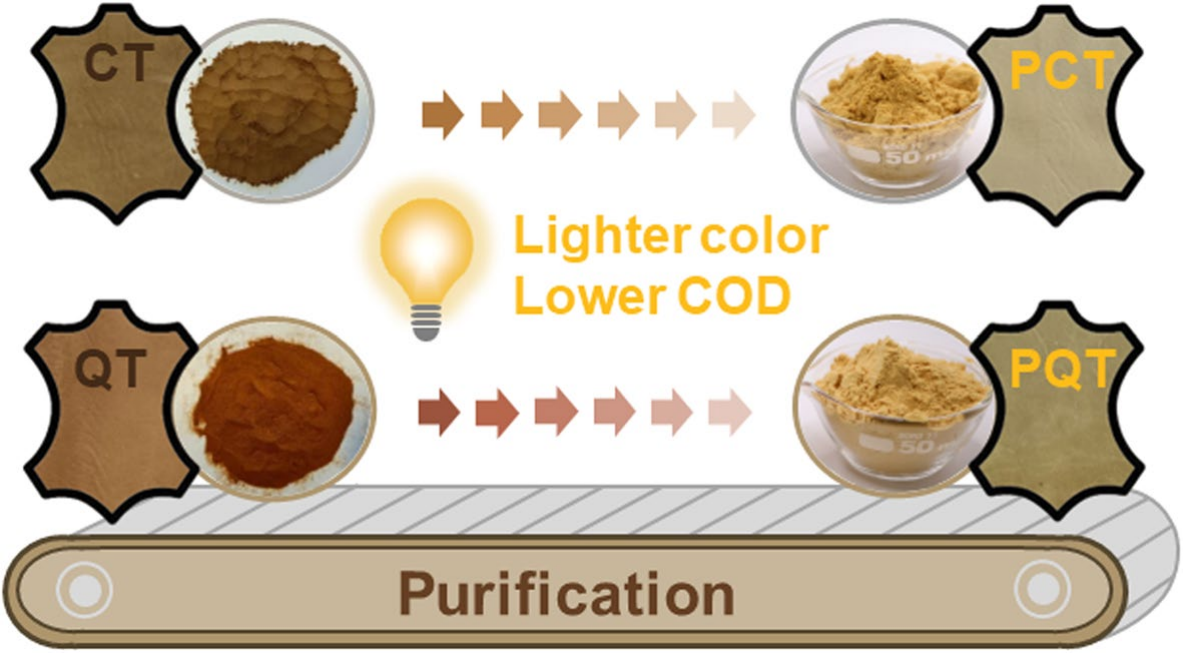

**Supplementary Information:**

The online version contains supplementary material available at 10.1186/s42825-024-00171-9.

## Introduction

Leather processing, one of the oldest activities of mankind, involves the transformation of food by-products (hides) into useful and valuable goods (leather) [1–4]. Throughout history, this fundamental skill has evolved into an intricate art form that produces everyday objects and valuable products for renowned fashion designers.

Currently, over 85% of the leather products are tanned with Cr(III) salts to produce “wet blue” due to its superior leather quality, cost-effectiveness, versatility, and replicability. The remaining 15% of the market employs alternative chrome-free metal salts (such as aluminium, titanium, or zirconium salts) or metal-free tanning agents like aldehydes, natural or synthetic tannins. These alternatives result in the production of chrome-free leather [5–8]. However, the use of chrome salts generates high quantities of chromium containing solid sludge, thereby leading to high disposal costs and environmental burden [4, 9–14]. Of every 1000 kg of salted bovine hides delivered to the tannery, only 250–300 kg is transformed into finished leather while the remaining 700–750 kg is discarded as waste, resulting in approximately 15–50 m^3^ of water effluents containing about 250 kg chemical oxygen demand (COD) and 100 kg biological oxygen demand (BOD) [15]. These statistics, coupled with stringent European regulations that prioritize environment protection, have gradually promoted the development and adoption of novel sustainable chrome-free and high exhaustion tanning systems for leather production [9, 13, 16]. Nevertheless, it should be noted that most chrome-free tanning agents also face increasing restrictions under REACH regulation due to their adverse impact on human health even though they don’t release chromiums. Moreover, the chrome-free leather usually exhibits inferior physical–mechanical properties [8]. Thus far, the choice between chrome and chrome-free leather involves complex technical, economic, and political considerations that continue to support chrome tanning as the dominant technology for leather production [5, 17].

In the past decade, vegetable biomass has emerged as a pivotal research topic in this field, as evidenced by numerous publications investigating its potential as tanning agents [18–23]. Vegetable tannins are regarded as a valuable alternative to chrome-free leather due to their natural origin and absence of hazardous substances. However, the utilization of vegetable tannins is currently limited due to the inferior physical and mechanical characteristics as well as higher production costs of vegetable-tanned leather. To enhance the tanning effects and improve the overall physical, mechanical, and organoleptic characteristics of vegetable-tanned leather, it is a common practice to combine vegetable tannins with aluminium salts for tanning. Although this approach improves hydrothermal stability and organoleptic properties of the resultant leather, it compromises the leather versatility and environmental sustainability [24–26]. The substantial amount of vegetable tannin used (up to 40 wt% based on weight of processed leather) negatively affects both the colour of tanned leather and COD in wastewater [27]. Thus, there is a great interest in exploring new processes that can simultaneously lighten the colour and enhance the tanning efficiency of vegetable tannin, mitigating their environmental impact on wastewater.

Therefore, in this work, chestnut tannin (CT) and sulfited quebracho tannin (QT) were subjected to a purification protocol to obtain purified tannins (PCT and PQT). These samples were characterised using gel permeation chromatography (GPC), nuclear magnetic resonance spectroscopy (NMR), Fourier-transform infrared spectroscopy (FT-IR), and high-performance liquid chromatography (HPLC). The tannin versus non-tannin ratio, pH, moisture and ash content were determined. Additionally, tanning trials were performed on depickled bovine pelts to investigate the tanning effects and environmental impacts of PCT and PQT.

## Experimental section

### Materials

The CT (chestnut tannin derived from *Castanea Sativa)* and QT (sulfited quebracho tannin derived from *Schinopsis Lorentzii*) were supplied by Silvateam (San Michele di Mondovì, Italy). The purified tannins (PCT and PQT), the residual fractions (RCT and RQT) and bovine depickled pelts were also supplied by Silvateam (San Michele di Mondovì, Italy). Solvents and chemicals were purchased from Sigma Aldrich (Italy) and used without further purification.

### Structural characterizations

#### Moisture content

The drying oven method is a thermogravimetric technique (loss on drying) in which the sample undergoes controlled desiccation for a specified duration of 4 h at a constant temperature of 102 °C ± 2 °C. The moisture content was determined by weighing the sample before (M1) and after (M2) drying, and subsequently calculating the ratio of weight loss (M1-M2) to the initial sample weight, according to Eq. 1:

1 $$M\;\left(\%\right)=\frac{M1-M2}{M1}\times100$$

#### Ash content

The samples (about 500 mg) were placed into a crucible and subjected to heating at 550 °C in a muffle furnace. The ash content (%) was determined by calculating the difference between the weight before (A1) and after (A2) heating, divided by the initial weight of the sample, according to Eq. 2:

2 $$Ash\;\left(\%\right)=\frac{A1-A2}{A1}\times100$$

#### Tannin content and pH value

The content of tannin and insoluble component in each sample was determined using the standard method (ISO 14088:2020).

The pH value was measured using a pH-meter (Hach Lange, mod. SensiON + PH 3) on a 10% dry matter colloidal solution.

#### GPC analysis

The number-average molecular weight (*M*_n_) of all samples was determined using an Agilent 1260 Infinity II chromatograph equipped with a UV detector (260 nm). A HP-PL gel 5 µm Mixed-D column protected with a PL gel 5 µm guard column (Agilent) was employed. Tetrahydrofuran (THF) served as eluent, and a calibration curve was established using polystyrene standards. The environmental included a flow rate of 1 mL/min and a column temperature of 30 °C. The extracts were derivatized according to a standard literature procedure prior the analysis [28].

#### NMR analysis

The ^1^H NMR and ^13^C NMR spectra were acquired using a Bruker UltraShield 400 spectrometer operating at frequencies of 400.0 and 101.0 MHz, respectively. Samples weighing precisely 100.0 mg were dissolved in 600 µL of deuterated dimethyl sulfoxide (DMSO-d_6_).

#### FT-IR analysis

The FT-IR spectra (1 wt% tannin/KBr) were recorded using a Perkin-Elmer Spectrum-One spectrophotometer, covering the range from 4000 to 450 cm^−1^ with 32 scans and a resolution of 4 cm^−1^.

#### HPLC analysis

The tannins were analysed by HPLC at 260 nm using a Perkin Elmer Flexar LC system, which consisted of the following parts: a Flexar PDA Plus Detector (diode array detector), Flexar Peltier LC Autosampler, Flexar Peltier LC Column Oven, a Flexar LC Quaternary Pump, and a Flexar Solvend Manger 5-CH Degaser. The analysis was performed on a Waters C18 XSelect® HSS T3 column (3.5 µm particle size, 4.6 mm × 150 mm) with an accompanying guard column (20 mm) made of the same material. Identification of different compounds was achieved using commercially available standards.

### Tanning tests

Tanning tests were carried out on depickled bovine pelts in 25 cm × 20 cm (approximately 300 g, with a thickness of 1.3–1.4 mm). The pelts were placed in a drum containing 100%wt water (based on the weight of pelts), NaCl (7°Bè), and 40%wt of tanning agents. Then, the hides were left to rotate overnight until reaching pH 4–5 (with basification using NaHCO_3_ if necessary). The tanned hides were dried, and their Ts values (IULTCS/IUP 16, 2015) were evaluated. A conventional post-tanning process was adopted to obtain the crust leathers.

### Physical–mechanical and organoleptic properties and environmental impact

The physical–mechanical properties (including thickness, tensile strength, tearing load, and elongation at break) of crust leathers were determined according to IUP 6 (ISO 3376:2020) and IUP 8 (ISO 3377–2, 2016). Organoleptic characteristics such as colour, fullness, resistance to UV and Xe light, as well as heat stability were assessed through manual and visual examination. Each property was assigned a score ranging from 1 to 5, with higher scores indicating better performance. The COD of wastewater after tanning processes was analysed using the APAT CNR IRSA 5130 method.

## Results and discussion

**Fig. 1:**
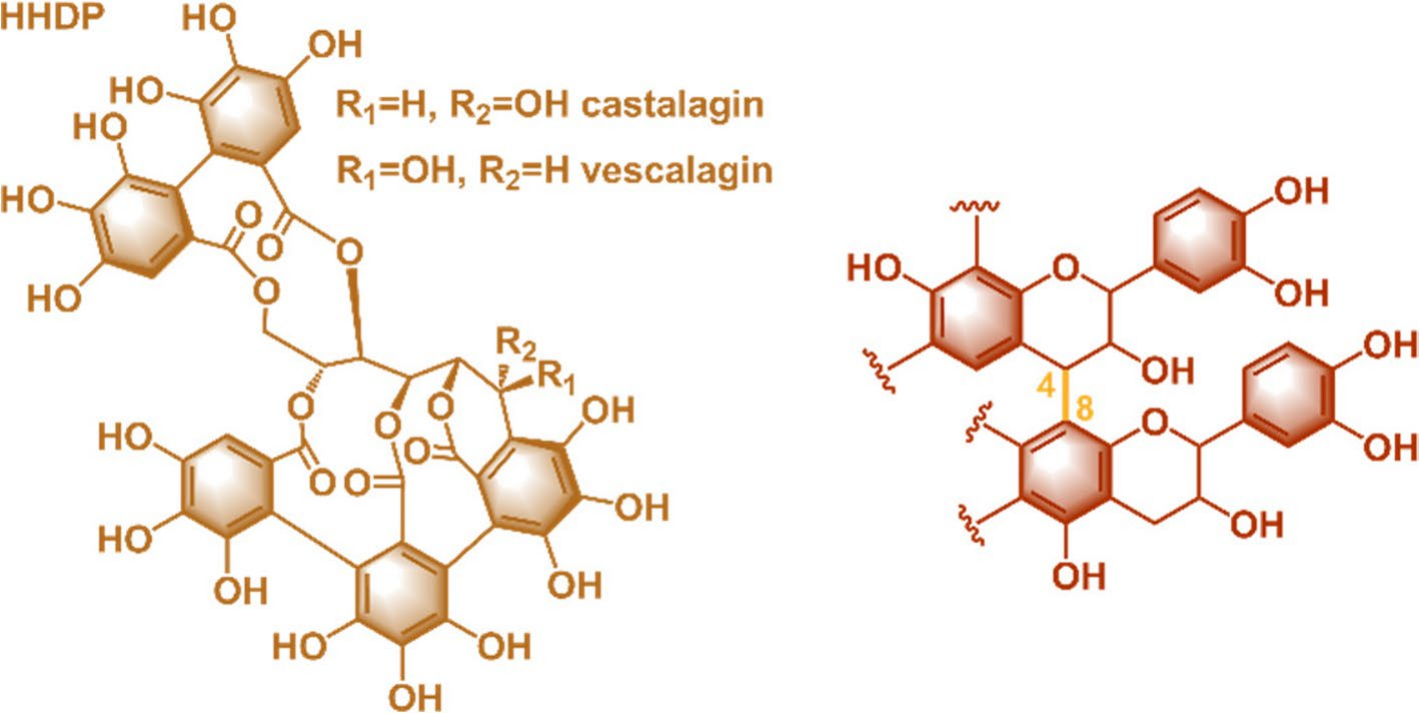
Main specie of chestnut and quebracho tannins

Vegetable tannins are naturally occurring water-soluble polyphenolic compounds found in various plant parts such as tree barks, stems, leaves, seeds, and roots, as well as nuts, fruits, spices, and herbs. These compounds typically have molecular weights ranging from 500 to 3,000 Da and can be mainly classified into hydrolysable tannins and condensed tannins [29–31]. Hydrolysable tannins consist of a carbohydrate core (*e.g.* glucose) esterified with gallic acid (gallotannins) or ellagic acid (ellagitannins), which can be easily hydrolysed in the presence of weak acids or bases. In contrast, condensed tannins (known as proanthocyanidins) are composed of flavonoid oligomers where the flavonoid units (mainly flavan-3-ols) are linked through C4-C6 or C4-C8 bonds (Fig. 1). Condensed tannin production accounts for over 90% of global commercial tannin output [29, 31]. This study analysed commercially available hydrolysable tannin extracted from *Castanea sativa* tree and modified condensed tannin derived from *Schinopsis Lorentzii* (Fig. 1).

Chestnut extracts are hydrolysable tannins comprising a mixture of castalin, vescalin, castalagin and vescalagin, along with other small molecules such as gallic acid and glucopyranose [32–34]. These ellagitannins are characterized by a glucose core esterified with at least one hexahydroxydiphenyl acidic moiety, which is formed through oxidative coupling between two gallic acid units [35].

**Scheme 1:**
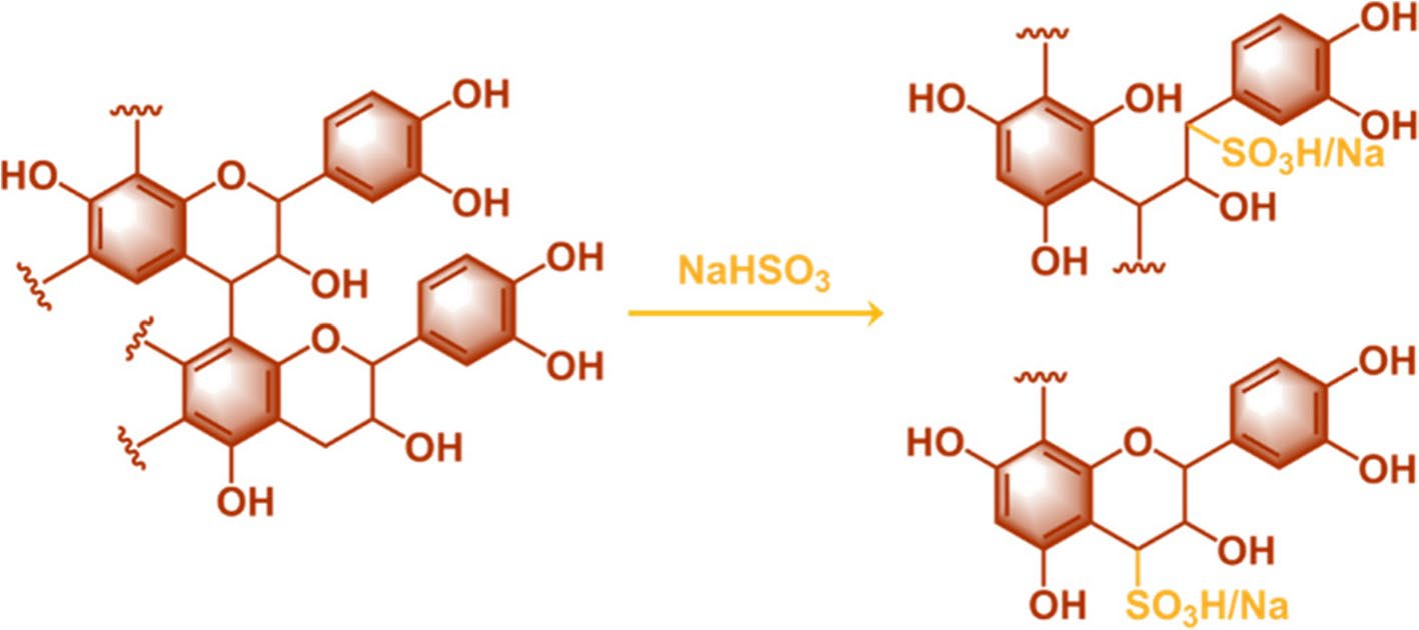
Sulfitation of quebracho tannin

Quebracho extracts are a mixture of proanthocyanidins (*i.e.* flavan-3-ol oligomers or high molecular weight polymers) and traces of smaller molecules such as ellagic or gallic acid [36, 37]. Due to their extremely low water-solubility, commercially available quebracho extracts are often sulfited using NaHSO_3_ to improve their solubility (Scheme 1) [38–41].

### Characterization of tannins before and after purification

#### Tannic content, pH, moisture, and ash content

**Fig. 2:**
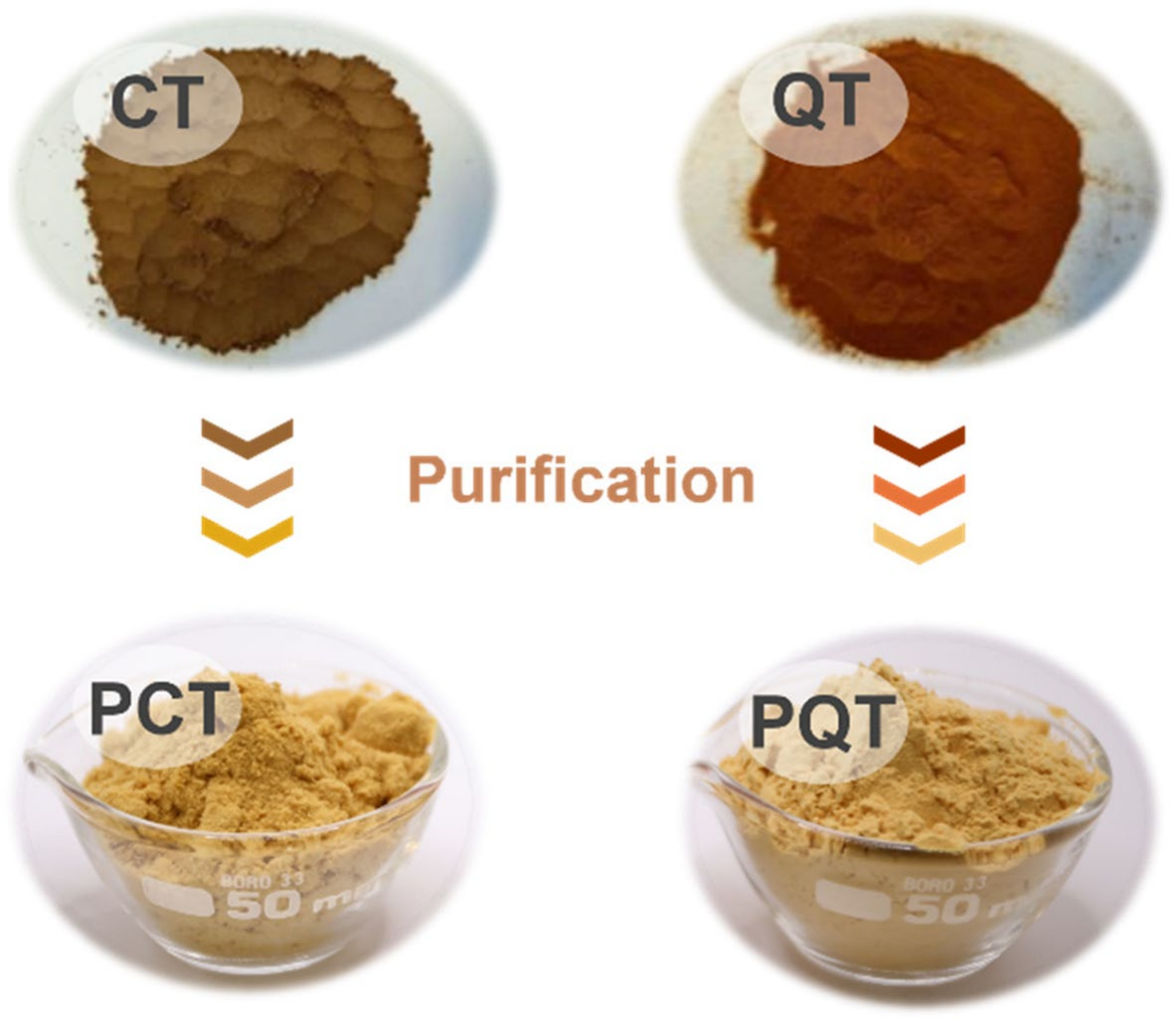
Powders of CT/PCT and QT/PQT

The CT and QT samples underwent a purification process to obtain the PCT and PQT fractions, as well as the residual RCT and RQT fractions. The tannin content, ratio of tannin to non-tannin (T/nT), moisture content, pH value, ash content, and *M*_n_ of CT, QT and their purified and residual fractions were determined and are presented in Table 1. The T/nT values of PCT and PQT were nearly twice compared to those of CT and QT, possibly owing to the reduced concentration of non-tannin molecules present in the purified fractions. In contrast, RCT and RQT showed remarkably low T/nT ratios and substantial ash contents because of the presence of abundant non-tannin molecules such as sugars and gallic acid. PCT and PQT exhibited relatively low moisture contents, which can be ascribed to the increased benzene rings with hydrophobicity. Meanwhile, the observed low pH values of the PCT and PQT may be attributed to the dissociation of phenolic hydroxyl groups. It is noteworthy that the PCT and PQT had a remarkably lighter colour compared to CT and QT (Fig. 2), which is expected to be highly beneficial for leather tanning. Regarding insoluble molecules, only negligible amounts were detected in all tannin extracts (< 1 mg/mL). The *M*_n_ of all samples fell within the range of 500–3,000, which is deemed suitable for leather tanning. Additionally, the colloidal solution of purified tannins was stored in a dark and cool environment, showing no formation of precipitates over a period of 4–6 months, thereby demonstrating their good stability.

**Table 1:** Chemical composition of CT and QT, as well as their purified and residual fractions

Sample	Color	Tannin content (%)	T/nT^a^	Moisture content (%)	pH	Ash content (%)	*M*_n_
CT	Brown	40.2	4.6	5.0	3.5	0.98	1,350
PCT	Yellow	83.1	8.3	4.0	3.3	1.60	680
RCT	Brown	52.0	1.2	4.3	3.6	5.06	530
QT	Red-brow	79.4	5.2	5.3	4.6	6.52	1,850
PQT	Yellow	87.8	11.2	3.9	4.5	3.90	780
RQT	Brown	54.3	1.4	4.5	5.2	29.13	650

^a^Ratio of tannin to non-tannin

#### NMR analysis

**Fig. 3:**
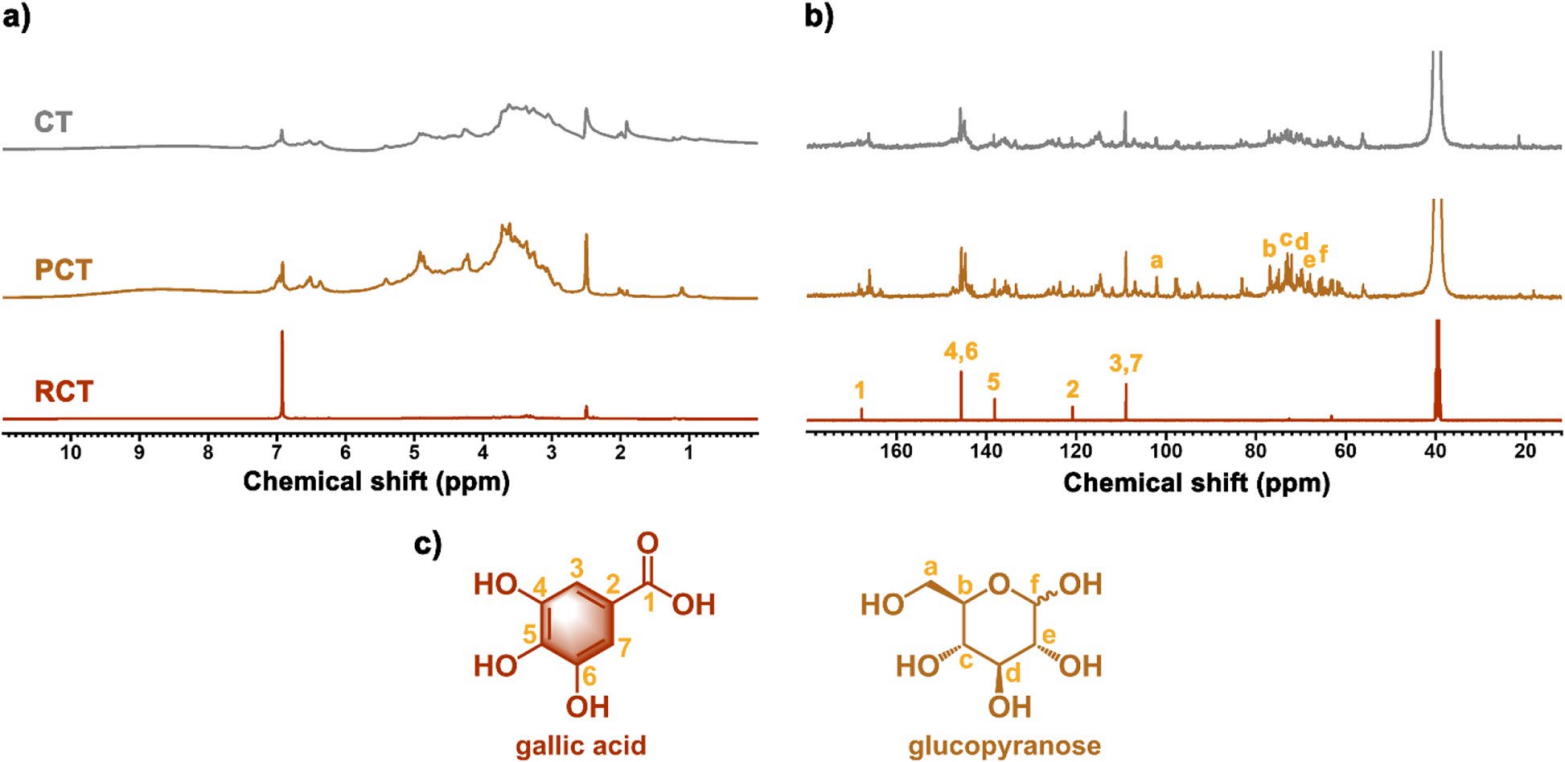
^1^H NMR (**a**) and.^13^C NMR (**b**) of CT and its purified (PCT) and residual fractions (RCT); The chemical structure of gallic acid and glucopyranose structure (**c**)

The chemical structure of CT and its purified and residual fractions was analysed using ^1^H and ^13^C NMR spectroscopy (Fig. 3). The ^1^H NMR spectra of CT can be divided into three main regions. Signals observed in the chemical shift range between 3.0 and 5.5 ppm were ascribed to the CH and CH_2_ groups, indicating the presence of carbohydrates such as glucopyranose and the aliphatic moiety of castalagin and vescalagin. The characteristic aromatic moiety of the polyphenols was detected between 6.0 and 7.5 ppm, while signals above 7.5 ppm corresponded to the broad -OH signal from phenolic compounds. Moreover, two signals at 6.93 ppm and 7.45 ppm were attributed to gallic acid and ellagic acid, respectively. The ^13^C NMR spectra also revealed three main sets of signals, in accordance with previous literature [32, 42]. Carbon signals ranging from 54.0 to 78.0 ppm corresponded to the glucopyranose ring and aliphatic carbons of castalagin and vescalagin. Signals between 100.0 and 150.0 ppm were ascribed to the aromatic carbon atoms. Notably, characteristic carbon signals related to hexahydroxydiphenyl and trigalloyl moiety were identified around 110 ppm. Aromatic carbon atoms bonded to the -OH functional group resonated between 140.0 and 150.0 ppm, together with carbonyl ester groups at around 170.0 ppm. These findings demonstrated CT is a typical ellagitannin.

In ^13^C NMR spectrum of PCT, glucopyranose signals were identified at chemical shifts of 101.9 (a), 76.8 (b), 73.3 (c), 69.9 (d), 68.4 (e) and 65.2 (f) ppm (Fig. 3) [32]. As for the residual fractions, RCT was mainly composed of gallic acid as evidenced by its characteristic peak at 6.93 ppm in the ^1^H NMR spectrum. These findings were further supported by corresponding peaks observed at chemical shifts of 167.8 (1), 145.6 (4,6), 138.1 (5), 120.8 (2), and 108.9 (3,7) ppm in its ^13^C NMR spectrum [43]. Interestingly, RCT, obtained as counterpart to PCT, mainly composed of gallic acid, thus confirming that PCT was obtained by the effective separation of lower molecular weight molecules (RCT) from CT through this purification process.

**Fig. 4:**
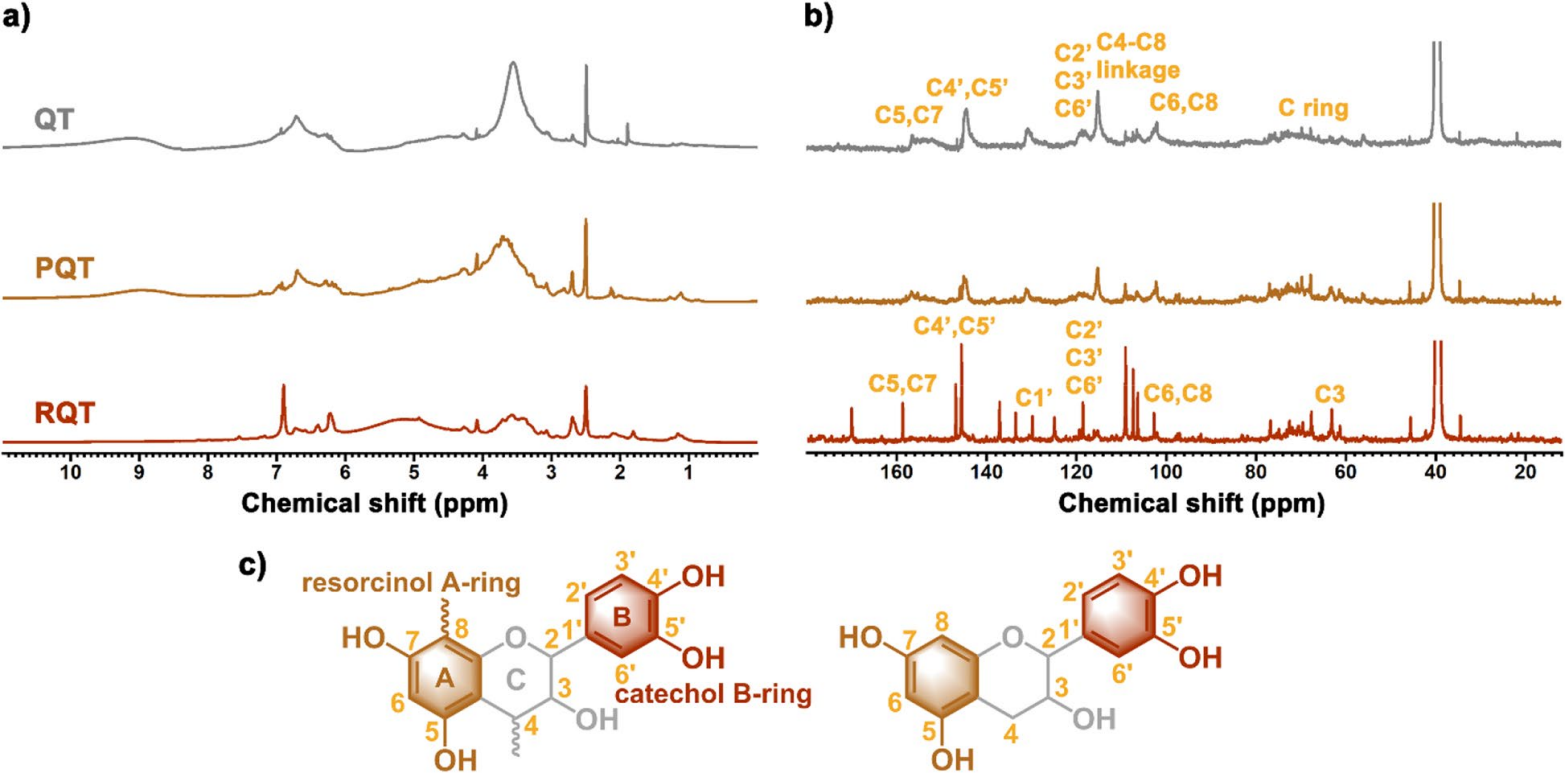
^1^H NMR (**a**) and.^13^C NMR (**b**) of QT and its purified (PQT) and residual fractions (RQT); The chemical structure of QT and catechin (**c**)

As for QT, the ^1^H NMR spectrum (Fig. 4) exhibited three main proton regions, indicating diverse proton types. Numerous signals were observed within the range of 2.9 to 5.7 ppm, corresponding to CH and CH_2_ groups attributed to the presence of carbohydrates ring (C ring). In the aromatic region (6.0–7.7 ppm), a broad peak was evident, representing aromatic protons of A and B rings, while between 8.4 and 10.5 ppm, a broad peak emerged due to the phenolic -OH functional group. In the ^13^C NMR spectra, all peaks have been assigned, confirming the flavonoid structure of QT predominantly composed of proanthocyanidins (about 95%) [36]. Specifically, signals in the chemical shift range of 61.0 to 77.0 ppm corresponded to the carbohydrate moiety (C ring), while those between 116.3 and 119.9 ppm and at 130.6 ppm (C2’, C3’, C6’ and C1’) together with the strong signal at 144.0 ppm (C4’, C5’) confirmed the presence of the catechol B-ring [44]. The strong signal at 115.1 ppm indicated the occurrence of a catechin dimer in QT extracts, as evidenced by the formation of a C4-C8 bond. The broad spectra bands observed between 150.0 and 160.0 ppm corresponded to C5 and C7 bonded to the -OH functional group, whereas the peak around 102.0 ppm (C6, C8) indicated the presence of the resorcinol structure of the A ring. Moreover, the signal at 55.8 ppm may be attributed to the methoxy groups present in lignin fragments [45, 46].

As shown in Fig. 4, the ^1^H and ^13^C NMR spectra of PQT exhibited more resolved peaks, indicating the effective removal of smaller molecules through the purification protocol. The presence of lower molecular weight substances in RQT fraction was confirmed by their highly resolved peaks in the ^13^C NMR spectra, which was further supported by average molecular weight analysis presented in Table 1. Notably, a characteristic peak at 115.1 ppm corresponding to oligomeric species was absent in RQT fraction, confirming their composition of lower molecular weight compounds such as gallic acid and catechin (158.8, 147.1, 129.5, 118.3, 102.8, 79.4, 63.1, 24.4 ppm) [43, 47].

In general, the NMR analysis confirmed the GPC data and provided additional information on the composition of each analysed fraction. In particular, the purification methodology demonstrated notable efficacy in removing small molecules such as gallic acid, glucopyranose, and catechin, thereby enriching oligomeric structures within vegetable tannins.

#### FT-IR analysis

**Fig. 5:**
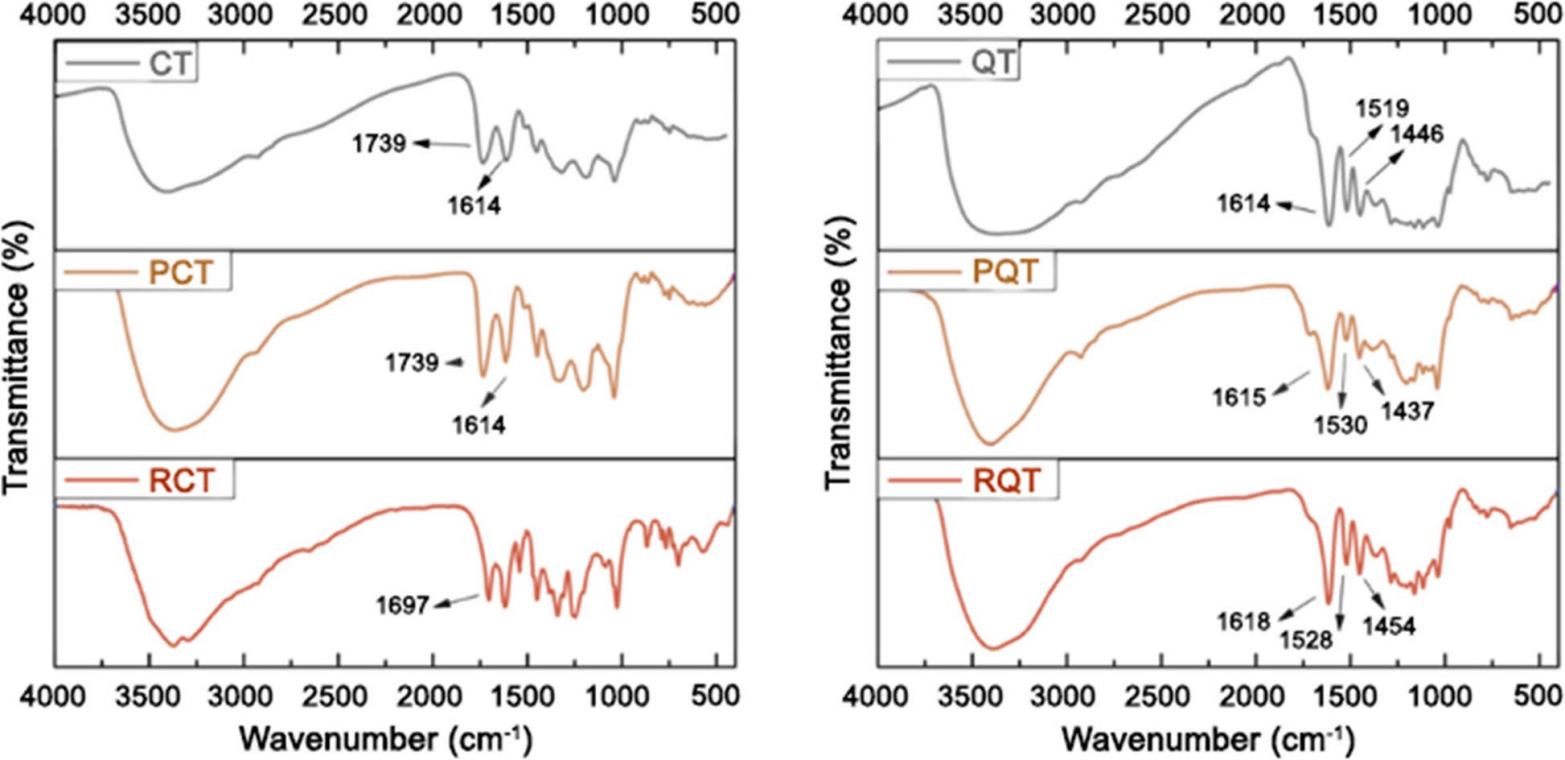
FT-IR spectra of CT (left) and QT (right), as well as their purified and residual fractions

The FT-IR spectra of CT and QT, as well as their purified and residual fractions, are presented in Fig. 5. In the FT-IR spectra of CT sample, within the 3400–3000 cm^−1^ region, the presence of OH stretching vibration was observed. Additionally, CH and CH_2_ stretching vibrations originating from aliphatic group in CT were identified around 2900 cm^−1^. A medium-strong band at 1739 cm^−1^, characteristic of hydrolysable tannins and attributed to the C = O stretching of esters derived from gallic acid, was evident in both CT and PCT spectra. Conversely, RCT spectrum exhibited typical bands associated with gallic acid, and specifically a signal at 1661 cm^−1^ corresponded to the C = O stretching. The region ranging from 1614 to 1450 cm^−1^ displayed similarities across all spectra and corresponded to the C = C aromatic stretching. As for the QT sample, the CH and CH_2_ stretching vibrations originating from the C-ring were observed around 2900 cm^−1^. The presence of the catechin moiety in both PQT and RQT was confirmed by strong bands observed at 1614 cm^−1^, 1519 cm^−1^, and 1446 cm^−1^, corresponding to the C = C aromatic stretching of condensed tannins [35]. Thus, FT-IR analysis further confirmed that the purification process did not modify the primary structure of tannin but only removed smaller molecules.

#### HPLC analysis

HPLC chromatogram of CT (Figure S1) confirmed the presence of low molecular weight substances, including castalagin, vescalagin, and gallic acid. Remarkably weak signal for gallic acid in PCT provided evidence for the effective removal of lower molecular weight compounds during the purification process. However, both castalagin and vescalagin were retained in PCT. HPLC chromatogram of QT exhibited greater complexity, with only gallic acid being identified (Figure S2). Nevertheless, it was observed that the purification process partially removed gallic acid for the much weaker signal of gallic acid in PQT.

### Tanning tests, physical and organoleptic properties of tanned leather

**Fig. 6:**
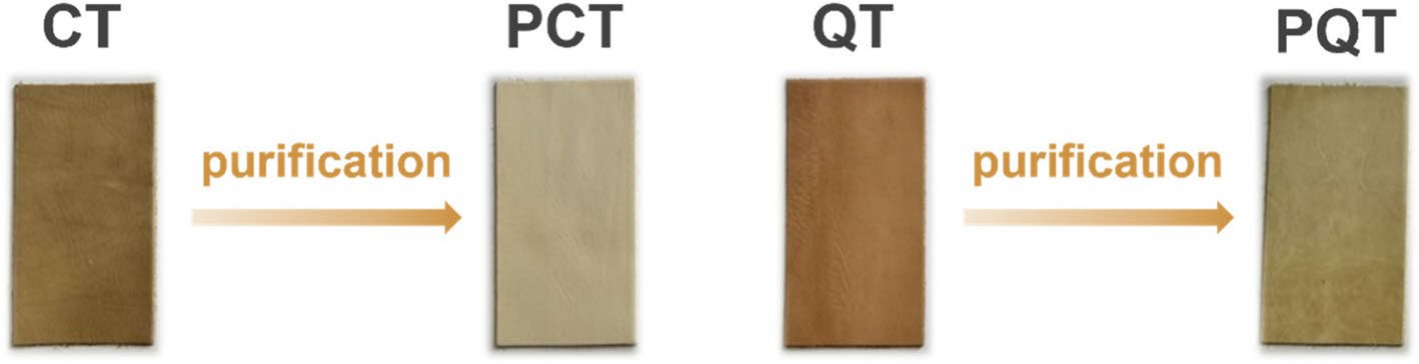
The colour of tanned leathers

The tanning effects of CT and QT, as well as their purified products (PCT and PQT), were investigated through tanning tests. As shown in Table 2, the tanning performance of PCT and PQT was found to be comparable to that of CT and QT in consideration of Ts values and physical mechanical characteristics of crust leathers, demonstrating that the purification process has little effect on the tanning properties of vegetable tannins. The tearing load of all the crust leathers exceeded the minimum requirement for upholstery leather, which follows a common rigid characteristic of vegetable tanned leather. However, the tearing load in PCT and PQT groups was lower compared to the CT and QT groups, indicating that these purified products may have enhanced softness of leather [9]. Notably, the purification process offers clear benefit by effectively reducing COD by 13.5% and 19.1% in wastewater from PCT and PQT tanning process respectively, probably due to the removal of lower molecular weight substances in vegetable tannins. Although further research is required to elucidate the tanning mechanism of different extracts reported in this study, it can be inferred that the PCT and PQT pose higher reactivity towards collagen, resulting in improved fixation of tannin in leather and thus reducing COD in wastewater. In addition, the crust leathers obtained using PCT and PQT exhibited noticeably lighter colour, which is conducive to the subsequent dyeing effect of leather (Fig. 6).

**Table 2:** Physical–mechanical, organoleptic properties of tanned leathers and COD in tanning wastewater

Sample	CT	PCT	QT	PQT	Upholstery leather
*Physical–mechanical properties*					
**Ts (°C)**	61 ± 2	57 ± 2	80 ± 2	78 ± 2	> 75
**Thickness (mm)**	1.23 ± 0.1	1.28 ± 0.1	1.42 ± 0.1	1.51 ± 0.1	
**Tensile strength (N/mm**^**2**^**)**	19.95 ± 0.9	13.25 ± 1.2	14.27 ± 1.4	11.80 ± 0.3	> 8
**Tearing load (N)**	245 ± 4	170 ± 7	202 ± 3	181 ± 4	> 40
**Elongation at break (%)**	20	18	29	24	< 80
*Organoleptic properties*					
**Colour**	3	4	3	5	
**Fullness**	4	5	4	5	
**Heat fastness**	4	3	2	2	
*Environmental impact*					
**COD (g/L)**	60.1 ± 0.8	52.0 ± 0.8	70.5 ± 0.8	57.0 ± 0.8

## Conclusions

The PCT and PQT samples, derived from CT and QT through purification process, were subjected to characterization using GPC, NMR, FT-IR, and HPLC techniques and subsequent tanning of depickled bovine pelts. The purification process effectively preserved the primary structure of the two tannins while selectively removing non-tannin compounds or smaller molecular components such as gallic acid, glucopyranose, and catechin. The leathers processed with PCT and PQT exhibited lighter colour but comparable organoleptic and physical–mechanical properties compared to those processed with CT and QT. Notably, the COD in wastewater by utilizing PCT and PQT as tanning agent was reduced, probably benefited from the removal of smaller molecules with poor fixation ability with collagen. Therefore, a rational purification approach for vegetable tannins can not only lighten the colour of vegetable-tanned leather but reduce the organic load in vegetable tanning wastewater, which is worthy of further research in the future. This study proposes a straightforward and convenient strategy to promote high-quality development of vegetable tannins.

## Supplementary Information

Supplementary Material 1.

## Data Availability

All data are included in this published article.

## Abbreviations

COD: Chemical oxygen demand
CT: Chestnut tannin
FT-IR: Fourier-transform infrared
GPC: Gel permeation chromatography
HPLC: High performance liquid chromatography
QT: Sulfited quebracho tannin
NMR: Nuclear magnetic resonance
PCT: Purified chestnut tannin
PQT: Purified sulfited quebracho tannin
RCT: Residual chestnut tannin
RQT: Residual sulfited quebracho tannin
