# Supplementary material for: Characterisation and tanning effects of purified chestnut and sulfited quebracho extracts
Source: Collagen Leather. 2024 Sep 2;6(1):28. doi: 10.1186/s42825-024-00171-9 (PMC11378616; doi:10.1186/s42825-024-00171-9)
Supplement: Supplementary file 1 — Supplementary Material 1. [file 42825_2024_171_MOESM1_ESM.docx]

**Supplementary Material**

**Characterisation and tanning effects of purified chestnut and sulfited quebracho extracts**

Silvia Conca^1^, Vanessa Gatto^1^, Riccardo Samiolo^1^, Samuele Giovando^2^, Andrea Cassani^2^, Elisa Tarabra^2^, Valentina Beghetto^1,3,4^*

^1^ Crossing S.r.l., Viale della Repubblica 193/b, Treviso, 31100, Italy.

^2^ CRCF Srl for Silvateam Spa, Via Torre 7, San Michele Mondovì, 12080, Italy

^3^ Department of Molecular Sciences and Nanosystems, University Ca’ Foscari of Venice, Via Torino 155, Mestre, 30172, Italy.

^4^ Consorzio Interuniversitario per le Reattività Chimiche e la Catalisi (CIRCC), via C. Ulpiani 27, Bari, 70126, Italy.

*Corresponding Author: [beghetto@unive.it](mailto:beghetto@unive.it)

**
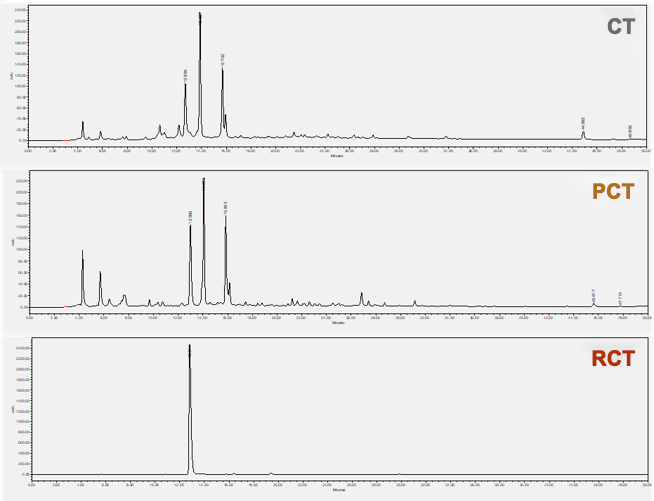
**

**Figure S1.** HPLC-DAD chromatograms of CT and its purified and residual fractions. (12.67 min, gallic acid; 13.88 min, vescalagin; 15.50 min, castalagin; 44.66 min, ellagic acid; 49.25 min, quercetin)


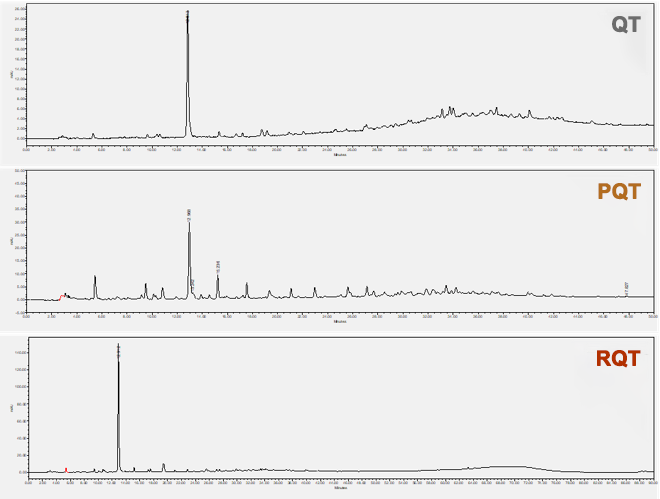


**Figure S2.** HPLC-DAD chromatograms of QT and its purified and residual fractions. (12.67 min, gallic acid; 13.88 min, vescalagin; 15.50 min, castalagin; 44.66 min, ellagic acid; 49.25 min, quercetin)
